# Editorial: Immune mechanisms of inflammation in NASH

**DOI:** 10.3389/fendo.2022.1039238

**Published:** 2022-09-21

**Authors:** Petra Hirsova, Xavier S. Revelo

**Affiliations:** ^1^ Division of Gastroenterology and Hepatology, Mayo Clinic, Rochester, MN, United States; ^2^ Department of Integrative Biology and Physiology, University of Minnesota, Minneapolis, MN, United States; ^3^ Center for Immunology, University of Minnesota, Minneapolis, MN, United States

**Keywords:** NASH, inflammation, T cells, neutrophils, LPS

## Introduction

Non-alcoholic fatty liver disease is highly associated with obesity and covers a wide spectrum of liver pathology ranging from a simple lipid accumulation to the more serious non-alcoholic steatohepatitis (NASH), characterized by inflammation, hepatocellular injury, and fibrosis (1, 2). Hepatic inflammation is primarily driven by innate and adaptive immune cells and is a critical component in the initiation and progression from simple steatosis to the more severe NASH (3). This *Research Topic* assembles review articles summarizing the latest research highlighting immune mechanisms of NASH with a focus on the innate and adaptive immune pathways that become activated and regulate disease.

## Endotoxins in the triggering of NAFLD

NAFLD is associated with increased intestinal permeability that facilitates the translocation of bacterial products into the liver which trigger hepatic inflammation. Kessoku et al. review the role of endotoxins in non-alcoholic fatty liver disease. Patients with NASH show an increased level of endotoxins, particularly lipopolysaccharides (LPS) produced by gram-negative bacteria. A compromised intestinal barrier and dysfunctional gut immune system allow intestinal-derived endotoxins to leak into the portal vein and reach the hepatic vasculature. In the liver, endotoxins activate hepatocytes and innate immune cells that initiate the inflammatory cascade and NASH progression. Another mechanism by which intestinal bacteria contribute to the progression of NASH is through short intestinal bacterial overgrowth (SIBO), a condition characterized by increased levels of microbes in the small intestine. SIBO can alter the absorption and metabolism of nutrients and contribute to the dysfunction of the intestinal barrier. Future studies are needed to determine if targeting endotoxins, or the microbiota, can be an option for the treatment of NASH.

## Neutrophils and T cells in the pathogenesis of NASH

Neutrophil infiltration is considered a hallmark of NASH and a key event in the triggering of inflammation. Hwang et al. review our current knowledge of the role of neutrophils in the pathogenesis of NASH and its resolution. Neutrophils are prominent producers of reactive oxygen species, proteases, and inflammatory mediators that cause hepatocyte injury, inflammation, and fibrosis. Neutrophil-derived myeloperoxidase and elastase, which mediate the production of reactive oxygen species and extracellular traps, have been shown to instigate NASH progression. Notably, lipocalin 2, a proinflammatory cytokine involved in obesity-related disease, can induce inflammation in crosstalk with hepatic macrophages. Although a pro-inflammatory role for neutrophils in NASH has been established, the heterogeneity and diverging functions of neutrophil subsets are less clear.

In addition to innate immune cells, it is now clear that adaptive immune cells such as T cells are important regulators in the pathogenesis of NASH. Hirsova et al. provide an update on the emerging roles of conventional and innate-like T cells in NASH. During disease progression, CD8 T cells acquire a non-specific hepatocyte killing activity, fueled by the fatty liver microenvironment. Interestingly, CD8 T cells may be also required for the resolution of NASH, suggesting functional heterogeneity. CD4 T helper cells, such as Th1 and Th17 cells, contribute to NASH pathogenesis through the secretion of their classical effector cytokines. Regarding innate-like T cells, several studies have focused on NKT cells, γδ T cells, or mucosal-associated invariant T cells, but their role in NASH is poorly understood.

## Regulation of the extracellular matrix in NASH and HCC

Non-alcoholic fatty liver disease and NASH represent an increasingly significant underlying cause of hepatocellular carcinoma (HCC), the most common primary liver cancer (4). Dashek et al. review the latest research focused on the regulation of inflammation and fibrosis by Reversion Inducing Cysteine Rich Protein with Kazal motifs (RECK) during the progression of NASH and HCC. In inflammatory conditions such as fibrosis, RECK can influence the remodeling of the extracellular matrix by regulating the activity of several matrix metalloproteinases. Notably, reduced RECK expression is a key event during HCC progression while its increased expression is associated with improved prognosis and overall outcomes. Although the precise mechanisms by which RECK downregulation, several micro-RNAs have been shown to target RECK during HCC. Thus, RECK is a promising prognostic marker for HCC and may be used as an indicator to discriminate immunotherapy candidates.

## Inflammatory mechanisms of hepatic stellate cell activation

During NASH, chronic hepatic inflammation leads to fibrosis because of excessive deposition of extracellular matrix by activated hepatic stellate cells (HSCs). Carter and Friedman review recent findings highlighting the crosstalk between hepatic immune cells and HSCs as a driving mechanism of liver fibrogenesis. Innate immune cells, such as macrophages and neutrophils, release several cytokines and pro-fibrotic factors that promote HSC activation. In turn, HSCs can signal back to the immune cells and amplify their proinflammatory activation, creating a feed-forward loop between inflammatory and fibrogenic pathways. Much less is known about the role of adaptive immune cells in the activation of HSCs but it is conceivable that T cells and B cells are involved in the profibrogenic processes. Importantly, the crosstalk between immune cells and HSCs is also required for the resolution of fibrosis in NASH. Future research is needed for a better understanding of the molecular mediators of these opposing processes. Precise identification of profibrotic and antifibrotic cell types and mediators could be then exploited for therapeutic strategies for NASH.

## Potential therapies for the treatment of NASH

There are no currently approved therapies for the treatment of biopsy-proven NASH. In this topic, Albhaisi et al. discuss the current and potential therapies targeting inflammation for the treatment of NASH. Although several drugs have been evaluated in clinical trials, results have been inconclusive or have shown adverse effects. Vitamin E improves steatosis and inflammation by reducing lipogenesis and oxidative stress but has been shown less effective at ameliorating hepatic fibrosis. Omega-3 polyunsaturated fatty acids have the potential to reduce oxidative stress, lipotoxicity, and inflammation and some data suggest that they may improve fatty liver disease, but not NASH. Other NASH therapies with inconclusive or insufficient evidence for its use include pentoxifylline, vitamin D, as well as inhibitors of caspases, components of the inflammasome, and vascular adhesion protein 1. Notably, inhibitors of the chemokine receptors 2 and 5 that are required for macrophage accumulation in tissues, were not successful at improving the severity of NASH in a recent phase IIb trial. To date, dietary modifications and weight reduction are the cornerstones for the management of NASH. However, combination treatments targeting both inflammation and metabolic disarrangements may offer effective therapy.

## Summary

Recent research efforts have exponentially increased our knowledge of the cellular and molecular mechanisms underlying the initiation and progression of liver inflammation in NASH. The activation of inflammatory pathways during NASH is a key event in the pathogenesis of the disease. However, emerging studies are revealing immune mechanisms regulating NASH resolution. Although B and T cells have received less attention, cells of the adaptive immune system are also key regulators of NASH. Advances in single-cell omics continue to help elucidate the functional relevance of immune subsets in disease progression as well as their crosstalk with other intrahepatic cells. Future studies are needed to clarify the precise mechanisms by which immune processes either drive disease progression or promote its resolution to guide the development of potential immuno-targeting therapies.

## Author contributions

All authors listed have made an equal, substantial, direct, and intellectual contribution to the work and approved it for publication.

## Funding

PH is supported by the Mayo Clinic Center for Biomedical Discovery and the National Institute of Diabetes and Digestive and Kidney Diseases (NIDDK, R01DK130884). XR is supported by grants from the NIDDK (R01DK122056) and the National Heart, Lung, and Blood Institute (R01HL155993).

## Conflict of interest

The authors declare that the research was conducted in the absence of any commercial or financial relationships that could be construed as a potential conflict of interest.

## Publisher’s note

All claims expressed in this article are solely those of the authors and do not necessarily represent those of their affiliated organizations, or those of the publisher, the editors and the reviewers. Any product that may be evaluated in this article, or claim that may be made by its manufacturer, is not guaranteed or endorsed by the publisher.
